# Strong strain dependence of friction in graphene kirigami allows engineering a negative coefficient of friction

**DOI:** 10.1073/pnas.2501728122

**Published:** 2025-07-11

**Authors:** Mikkel Metzsch Juel, Anders Malthe-Sørenssen, Henrik Andersen Sveinsson

**Affiliations:** ^a^The Njord Centre, Department of Physics, University of Oslo, Oslo 0316, Norway; ^b^Institute for Geotechnical Engineering, Department of Civil, Environmental and Geomatic Engineering, ETH Zürich, Zürich 8093, Switzerland; ^c^Swiss Federal Institute for Forest, Snow and Landscape Research Institute for Snow and Avalanche Research, Davos Dorf 7260, Switzerland

**Keywords:** friction, metamaterials, negative friction coefficient, kirigami, commensurability

## Abstract

The friction force typically increases linearly with normal load with a constant of proportionality called the coefficient of friction. Most materials exhibit a positive friction coefficient, so that an increase in the normal load leads to an increase in the friction force. Recently, materials with negative friction coefficients have been observed at meticulously constructed interfaces due to an interplay between superstructures at heterojunctions, out-of-plane buckling, or the ordering of thin water films. However, the magnitude of the negative friction coefficient is typically much less than $10^{-2}$, the geometries are highly restrictive, and the mechanisms are difficult to scale to larger systems. Here, we show that a friction coefficient of −0.08 can be obtained for a graphene sheet modified with kirigami-inspired cuts when inducing sheet tension through a normal load coupling. We use molecular dynamics simulations to show that the frictional behavior of kirigami cut sheets exhibits a strong nonmonotonic dependence on in-plane strain, while only weakly influenced by normal loading. We argue that the strong influence of strain arises from changes in commensurability between the graphene sheet and the substrate when the sheet deforms and buckles out of plane. We propose a simple nanomachine design that couples normal loading and sheet tension which enables the realization of different frictional laws, including a negative coefficient of friction. This represents a unique approach to creating tunable frictional surfaces and opens up applications in sheet-like systems across scales.

## Significance

Frictional force on bodies in relative motion typically increases with the normal load, indicating a positive friction coefficient. Systems with negative friction coefficients have been demonstrated but only with low magnitudes. Using molecular dynamics simulations, we here demonstrate a method to achieve high-magnitude negative friction coefficients. By applying kirigami patterns to a graphene sheet sliding on a silicon substrate and introducing a nanomachine coupling between normal load and sheet tension, we induce a commensurability effect, creating an increase–decrease–increase relationship between the normal load and friction force. This frictional behavior is susceptible to both the nanomachine coupling and the kirigami patterning, offering a wide range of design options, opening for new classes of metamaterials with complex and tunable friction properties.

The frictional force $F_{\text{f}}$ between two bodies in relative motion typically increases with increasing normal load $F_{N}$. When the relationship between the friction force and the normal load is linear, the friction coefficient is conventionally defined as $\mu =F_{\text{f}}/F_{\text{N}}$. In the case of a nonlinear relationship, a differential friction coefficient can be defined as $\mu \left(F_{\text{N}}\right)=\text{d}F_{\text{f}}/\text{d}F_{\text{N}}$ (1), representing the local slope of the friction–normal load relationship. In contrast to the conventional friction coefficient, the differential friction coefficient can take negative values, corresponding to a decreasing magnitude of the friction force with increasing load, with the restriction that the friction force itself can never become negative. From this point onward, we adopt the differential friction coefficient as our working definition.

Negative friction coefficients have previously been obtained under the retraction of a diamond atomic force microscopy tip sliding on a graphite surface (2), and at various heterojunctions (3–6). At graphite–boron nitride heterojunctions (3), the negative friction coefficient is attributed to an interplay between the moirÃ© superstructure of the heterojunction, out-of-plane buckling, and the applied load. At graphite–mica heterojunctions (4), the negative friction coefficient arises due to an even more intricate process due to increased ordering of a thin water film, at the graphite–mica interface, under increased load. A negative friction coefficient has also been predicted for a heterojunction where a boron nitride sheet slides on polycrystalline graphene (5), and recently also demonstrated for a heterojunction where a graphite sheet slides on aluminum oxide (6), but in this case by a mechanical edge effect. However, only some studies (4, 6) show a negative friction coefficient both under loading and retraction, the graphite–boron nitride system is very sensitive to humidity, and the magnitude of the negative friction coefficient has so far been less than $10^{-2}$. Thus, new mechanisms may be needed to generate systems that robustly produce negative friction coefficients of higher magnitude.

A possible path to develop controllable frictional properties is through commensurability. Simplified chain models such as the Frenkel–Kontorova models (7, 8) predict a state of zero static friction and a highly reduced kinetic friction, known as superlubricity. In these models, superlubricity is a result of an incommensurable state, i.e., when atoms in the two interacting chains are generally misaligned, such that periodic forces from atomic lattices cancel out over longer distances. This invites the idea of controlling the friction response by tuning the degree of commensurability.

The commensurability between two surfaces can in principle be tuned by designing metamaterials, where material compositions are meticulously organized to enhance certain physical properties (9–14). This is often achieved either by intertwining different material types or removing certain regions completely. Due to the impracticality of synthesizing material samples for real-world exploration of novel metamaterials, numerical methods offer a cost-effective alternative to testing numerous designs. In recent papers by Hanakata et al. (9, 10), numerical studies have showcased that mechanical properties of a graphene sheet, specifically yield stress and yield strain, can be altered through the introduction of kirigami-inspired cuts into the sheet. Kirigami extends from the well-known origami by including cutting of the sheet in addition to folding. Hanakata et al. (9, 10) attribute the variation in mechanical properties to the nonlinear effects arising from the out-of-plane buckling of the sheet when strained. Numerical methods, such as molecular dynamics (MD), have been widely used for the study of nanoscale friction (15–24) and lately the use of kirigami structures as a means of controlling friction has attracted growing interest (25–27). Moreover, previous experimental and numerical studies have shown that axial strain can lead to a reduction in the frictional force of a graphene sheet (23, 28).

Here, we use molecular dynamics simulations to demonstrate that we can obtain a strong nonmonotonic friction–strain relationship by introducing kirigami cuts in a graphene sheet and that this relationship can be exploited to obtain negative friction coefficients when coupling sheet tension with the normal load. In our simulations, the mere introduction of kirigami cuts or simply straining a noncut sheet, does not significantly change the frictional state of the sheet. However, when combining kirigami cuts with sufficient strain, the commensurability between the graphene sheet and the silicon substrate changes, superlubricity breaks, and friction increases dramatically. We show that for two different kirigami cut patterns, the friction–strain relationship is characterized by an increase–decrease–increase relationship with increasing strain. We finally introduce simulations where we couple the normal loading and the sheet tension on the kirigami-patterned graphene sheet to demonstrate how the friction–strain relationship in this system can be exploited to construct a nanomachine displaying a negative friction coefficient in both the loading and unloading stages.

## 1. Molecular Dynamics Simulations

**Fig. 1.**
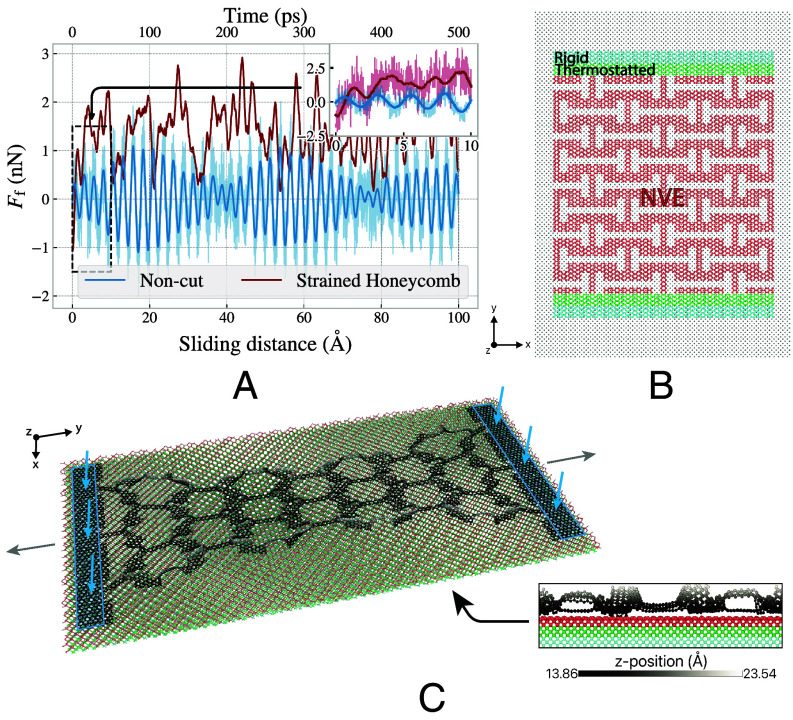
Overview of friction simulations in the graphene sheet–silicon substrate system. (*A*) The friction force $F_{\text{f}}$ versus sliding distance for an intact (noncut) graphene sheet (blue) and a highly strained (ε=0.71) Honeycomb-patterned sheet (red). Both sheets were subjected to a normal load of 1 nN. The force trace values have been smoothed with a fifth order Savitzky–Golay filter with a 15 ps time window. Raw force traces only subjected to a 0.1 ps running average are shown more transparently in the background for reference. The *Inset* shows the first 10 Å of sliding, particularly showing the high-frequency oscillations of the superlubric, intact sheet. (*B*) *Top*-view of an unstrained Honeycomb-patterned graphene sheet on a silicon substrate. The sheet atoms are colored to indicate the NVE (red), thermostated (green), and rigid (blue) regions of the sheet, and the substrate atoms are colored in gray. (*C*) Perspective and side view (*Inset*) of the system, showing the strained state of a Honeycomb-patterned graphene sheet. The substrate coloring in (*C*) is equivalent to the sheet coloring in (*B*), whereas a grayscale colormap shows z-position of particles in the graphene sheet to visualize out-of-plane buckling. The blue arrows and boxes mark the regions where the normal load is applied.

We constructed two types of kirigami-patterned graphene sheets for molecular dynamics (MD) simulations as seen in Fig. 5: a Tetrahedron pattern and a Honeycomb pattern, named for their out-of-plane buckling shapes. These designs were inspired by common macroscale patterns (29) and (30) respectively. The simulated system consists of a 130Å×163Å single layer of graphene in contact with a crystalline silicon substrate of appropriate size. Fig. 1 *B* and *C* visualizes the sheet-substrate system with the Honeycomb pattern. The system is further divided into three types of regions as marked in Fig. 1 *B* and *C*: NVE, i.e., conservative dynamics (red), thermostated (green) using a target temperature of T=300K and rigid (blue). The combined thermosttated and rigid regions of the sheet constitute the handling regions which are used for straining, loading, and sliding the sheet. The NVE region of the sheet serves as a canvas for the kirigami pattern. The interatomic forces in the graphene sheet (C–C) are modeled with a Tersoff potential (31), the silicon substrate (Si–Si) is modeled with the Stillinger–Weber potential (32), and the forces between the graphene sheet and the silicon substrate (C–Si) are modeled using a Lennard–Jones potential. The simulation cell has periodic boundary conditions in the x and y directions. The sheets were subjected to the following procedure to examine their frictional behavior under various normal loads and strains: After a short relaxation period of 15 ps the graphene sheet is strained by a linear translation of the handling regions in the ±y direction with a strain rate of 0.01 ps-1 until a specified uniaxial strain level is reached. The fully strained sheet is then loaded uniformly on both handling regions, normal to the sheet and substrate, with a specified normal load $F_{\text{N}}$. We chose to apply strain and normal load only to the handling regions to remain consistent with our nanomachine design and to avoid constraining out-of-plane buckling in the kirigami-patterned area. Finally the sheet is slid in the positive y-direction by translating the handling regions at a constant speed of 20 ms^−1^ for 2 ns, so that it slides 40 nm. The choices regarding temperature, sliding speed, and simulation timestep are based on a systematic investigation detailed in ref. 33. If a C–C bond in the graphene sheet breaks during a simulation, this is flagged as a rupture event and the simulation is halted. The simulation procedure is implemented and carried out using LAMMPS (34). More details of the simulations are provided in *Materials and Methods* and as input scripts (35).

## 2. Strain-Dependent Friction

**Fig. 2.**
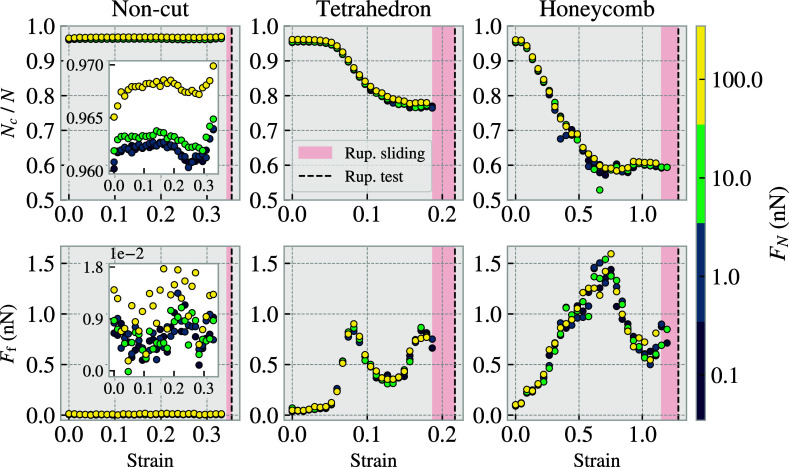
Relative contact $N_{c}/N$ (*Upper* row) and friction force $F_{\text{f}}$ (*Lower* row) for independent MD simulations at various sheet strains and normal loads. $N_{c}$ is the number of sheet atoms in contact with the substrate, defined by a 4 Å distance threshold, which is scaled by the total number of atoms in the sheet N. The columns correspond to the applied kirigami patterns. 30 strain values are sampled uniformly between zero and their respective rupture strain (black dotted line) computed at zero normal load. The red shaded area denotes the strain range for which simulations where excluded due to rupture events in sliding simulations. The normal load values are 0.1, 1, 10, and 100 nN denoted by the marker colors. Both the contact and friction values are averaged over the last 1 ps of sliding.

From the molecular dynamics simulations of graphene sheets on silicon substrates, we measure the kinetic friction $F_{\text{f}}$, as the force acting from the substrate on the sheet during sliding. Fig. 1*A* shows a friction force trace for a noncut and a strained Honeycomb-patterned sheet showcasing significant oscillations in the force readings. The noncut sheet appears to exhibit periodic behavior. Fourier analysis reveals that the two strongest oscillations have periods of 2.62±0.02Å and 71±15Å. The 2.62±0.02Å period might be ascribed to stick–slip motion on the scale of the nearest neighbor distances within the sheet (1.42 Å) and the substrate (2.35 Å). The 71±15Å period might be attributed to a resonance phenomenon. Since friction is typically strongly correlated with the real contact area, we measure the number of atoms in contact $N_{c}$, scaled by the total number of atoms in the sheet N. We define contact by a sheet-substrate distance threshold of 4 Å corresponding to roughly 120% of the Lennard–Jones equilibrium distance. To account for the oscillatory behavior observed in Fig. 1, and the time required to reach steady state sliding, we report the force and contact number as averages taken over the final half of the sliding phase, i.e., the last 1 ns of the simulation. Fig. 2 displays the resulting average relative contact $N_{c}/N$ and friction force $F_{\text{f}}$ for simulations at different sheet strains and normal loads. In the case of the kirigami sheets, contact decreases monotonically with strain. However, for both kirigami patterns, the friction force exhibits an increase–decrease–increase behavior with strain. We therefore conclude that in our kirigami system, the contact area cannot explain the friction force trend. This is in contrast to the expected behavior from asperity theory, where friction is expected to be proportional to the real contact area (36). For the noncut sheet, we do not find any significant dependence on strain. Thus we attribute the difference in behavior to the kirigami patterning. We observe that a three-orders-of-magnitude increase in the normal load, corresponding to a stress range of ∼[0.47MPa,470MPa], results in only a small change in friction, which is negligible in comparison to the variations of friction with strain, seen for the kirigami sheets.

## 3. Negative Friction Coefficient

**Fig. 3.**
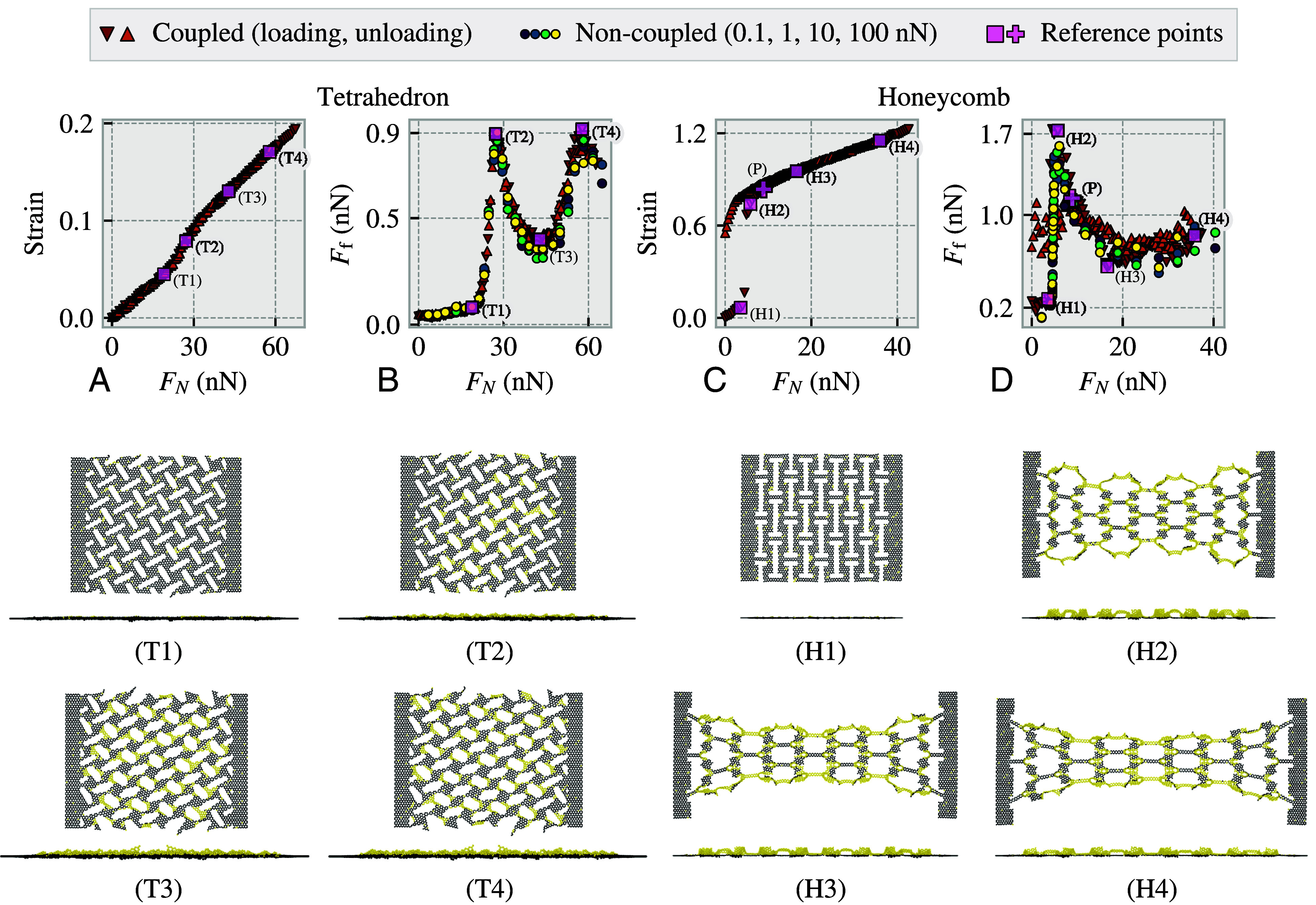
Frictional behavior for a coupled system, where the sheet strain is controlled by a tension force $F_{t}$, modeled as $F_{t}=T\cdot F_{N}$, for normal load $F_{N}$ and a load-to-tension ratio T=1. We perform independent simulations at uniformly spaced load values, using a loading rate of 0.01 nNp^−1^s and measuring the average friction over the last 1 ns of sliding. We perform a similar routine for simulations first loaded to a load value close to the recorded rupture values and then unloaded toward uniformly sampled values. Panels (*A* and *B*) and (T1)–(T4) relates to the Tetrahedron pattern while panels (*C* and *D*) and (H1)–(H4) relates to the Honeycomb pattern. (*B* and *D*) illustrates the friction–load curve for the coupled system. The red upward-pointing triangles represent results obtained under increasing load, while the orange downward-pointing triangles represent results under decreasing load, indicating the presence of a hysteresis effect. The circular markers correspond to the result of the noncoupled system in Fig. 2 where the x-axis is mapped from strain to load values using an interpolation based on the coupled system strain–load relationship. Their original normal loads are indicated by the marker colors inheriting from Fig. 2. (*A* and *C*) shows the coupled system strain–load curve. In both plots, the pink squares relates the sheet position states, (T1)–(T4) and (H1)–(H4), to points on the strain–load and friction–load curves in the loading phase. (T1)–(T4), (H1)–(H4) are showing a *Top* view (*Upper* part) and a side view (*Lower* part) of the sheet-substrate system. The substrate atoms are omitted while the sheet atoms are colored black for the atoms in contact with the substrate and yellow for noncontacting atoms.

We propose that the strong nonmonotonic friction–strain relationship for the kirigami sheets observed in Fig. 2 can be exploited to create a system with negative friction coefficients. Given that the influence of strain on friction significantly outweighs that of the normal load, a coupling of strain and normal load can produce a friction–load curve with a trend similar to the friction–strain curve in Fig. 2. Using the differential friction coefficient definition, $\mu \left(F_{N}\right)=\text{d}F_{\text{f}}/\text{d}F_{\text{N}}$, such a trend corresponds to a negative friction coefficient for certain load ranges. We suggest that a friction–strain coupling can be achieved by creating a nanomachine setup which mechanically couples the loading force $F_{N}$ to a sheet tension force $F_{t}$. We do not attempt to model the specifics of such a nanomachine here, but we assume instead a simple linear coupling, $F_{t}=T\cdot F_{N}$, with a load-to-tension ratio T=1. This might resemble a triangular structure where the sheet constitutes the bottom face and the load is applied at the apex. We model the coupled system using the same setup as before, but we now allow the handling regions to move relative to each other. The sheet strain is determined by the equilibrium between the internal forces in the sheet and the externally applied sheet tension force, dictated by the normal loading. We conduct a series of individual simulations for various normal loads, equivalent to various sheet tensions. From these simulations, we measure the resulting strain and friction force on the sheet, as shown in Fig. 3*A*–*D*. The normal load–strain curves, (*A* and *C*), show a monotonic increase in strain with normal load. In the Tetrahedron system, this relationship is nearly linear, with only a small change in slope when the sheet starts to buckle. However, in the Honeycomb system, the strain increases drastically from approximately 0.08 to 0.7 at a load of $F_{N}∼4.7\;\text{n N}$, which is associated with a cascading buckling of the Honeycomb structure from state (H1) to (H2) shown in Fig. 3. In panels (*B* and *D*) we show the normal load–friction curves for the coupled systems in comparison to the results from the noncoupled system (Fig. 2). To allow for this comparison we map the noncoupled data points from strain to normal load using the strain–load relationship of the coupled system seen in Fig. 3 *A* and *C*. We find good visual consistency between the load–friction curves of the coupled and noncoupled simulations, indicating that the friction force–strain relationship in Fig. 2 is retained also when the system is strained and loaded simultaneously in a coupled fashion. For the Honeycomb pattern, we observe some hysteresis effect upon unloading the system beyond the point (P), likely caused by a barrier associated with the abrupt transition from (H1) to (H2). For both kirigami patterns the loading and unloading stages of the friction–load curves contain a convex part, (T2)–(T4) and (P)–(H4) leading to a negative friction–load slope approximately from (T2) to (T3) for the Tetrahedron pattern and from (H2/P) to (H3) for the Honeycomb pattern. By considering the maximum and minimum friction values in these ranges in the loading stage, we compute the following average negative friction coefficients:

$$\begin{array}{ccc} & \underset{\text{(strain:}\;0.08\to 0.13\text{)}}{\text{Tetrahedron:}}\;\mu_{\text{neg}}∼\frac{0.38\;\text{nN}-0.90\;\text{nN}}{43.53\;\text{nN}-27.22\;\text{nN}}=-0.03, & \\ & \underset{\text{(strain:}\;0.84\to 0.95\text{)}}{\text{Honeycomb:}}\;\;\mu_{\text{neg}}∼\frac{0.56\;\text{nN}-1.15\;\text{nN}}{16.60\;\text{nN}-8.97\;\text{nN}}=-0.08. \end{array}$$

This serves as a proof of concept that substantially negative friction coefficients can be reached by the combination of a load-strain coupling and the introduction of kirigami cuts into a graphene sheet. Considering that the friction force is strongly dependent on the strain, with negligible dependence on the normal load, we expect the magnitude of the friction coefficients to scale with the load-to-tension ratio T, i.e., $\mu_{\text{neg}}\propto T$. In general, the specific friction–load curve is anticipated to be controlled both by the nanomachine coupling design, through the load-to-tension coupling, and by the kirigami sheet pattern dictating the tension–strain and strain–friction relationship. This leads to the following chain of influence:

$$\begin{array}{c} \begin{array}{ccccccc} \text{load} & ⟹ & \text{tension} & ⟹ & \text{strain} & ⟹ & \text{friction}\\ & \begin{array}{c} \text{nanomachine}\\ \text{coupling} \end{array} & \; & \text{kirigami} & \; & +\begin{array}{c} \text{kirigami}\\ \text{substrate} \end{array} \end{array} \end{array}$$

## 4. Commensurability

**Fig. 4.**
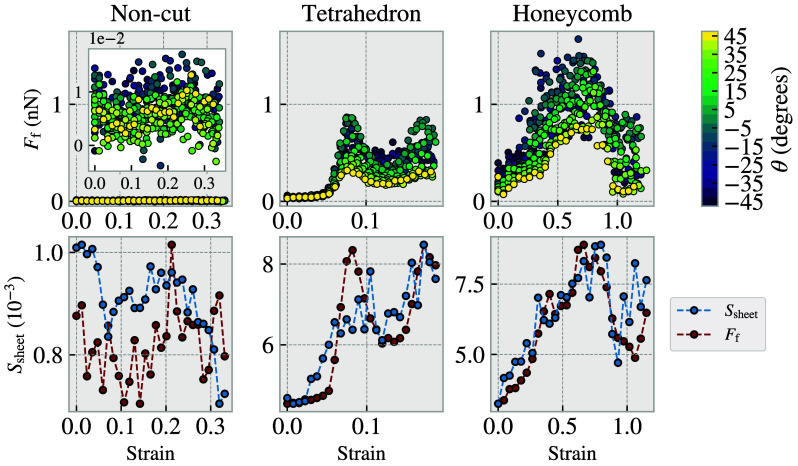
Investigation of the influence of commensurability. *Top* row: Friction–strain relationship with varying sliding orientation θ as indicated by the marker colors. $\theta =0^{{}^{\circ}}$ corresponds to the default sliding orientation (positive y-direction) used in all other simulations. *Bottom* row: Commensurability S versus sheet strain (blue, *Left* y-axis) in comparison to the friction–strain trend (red, no y-axis) from Fig. 2 using a normal load of 1 nN. The friction force is simply min-max scaled to align with the commensurability data.

It has been shown both experimentally, for graphite on graphite (37), and numerically, using a tight-binding atomistic simulation for graphene on graphite (38), that the kinetic friction varies with the relative orientation and sliding direction between the sheet and the substrate. This phenomenon has been attributed to a change in commensurability between the sheet and the substrate. Such sensitivity to commensurability has also been reported for MD simulations (16, 22, 24) and further supported by experimental measurements of interaction energies for graphite on graphite (39). The commensurability dependence also shows up in relatively simple 1D chain-like models such as the Frenkel–Kontorova (7) and Frenkel–Kontorova–Tomlinson (8) models. Dong et al. (40) have shown that a 2D extension of the Frenkel–Kontorova–Tomlinson model yields an additional dependence between the friction response and the sliding path through the modeled substrate potential. However, this well-established theory only applies to intact sheets. Upon visually inspecting the strained kirigami sheets depicted in Fig. 3, it is evident that the out-of-plane buckling allows for changes in the sheet’s surface and local orientation, which could potentially lead to changes in commensurability. This allows for both increasing and decreasing commensurability during straining, resulting in a nonmonotonic friction–strain relationship. To evaluate this hypothesis, we first performed a series of simulations using the same procedure as for the simulations underlying Fig. 2. In this case, however, we maintained a normal load of 1nN but instead varied the sliding orientation, θ, of the sheet relative to the substrate, as shown in the *Top* row of Fig. 4. We observe that the friction–strain relationship for the kirigami sheets is modified by the orientation, with some intervals amplifying the effect of the strain, and some intervals reducing it, which supports the commensurability hypothesis. In order to directly quantify the commensurability, S, we follow the approach of refs. 41 and 42, using the normalized sum

[1] $$S=\sqrt{\frac{{\left|\sum_{j=1}^{N}e^{i\text{G}\cdot {\text{r}}_{j}}\right|}^{2}}{N^{2}}},$$

where G is the sum of the reciprocal lattice vectors of the substrate, and ${\text{r}}_{j}$ is the position vector of the j-th atom in the sheet, consisting of N atoms. For substrate primitive lattice vectors ${\text{a}}_{i}$ and corresponding reciprocal vectors ${\text{b}}_{i}$ we have $\text{G}={\text{b}}_{1}+{\text{b}}_{2}+{\text{b}}_{3}$. Geometrically, the exponential term in Eq. **1**, $e^{i\text{G}\cdot {\text{r}}_{j}}$, maps the position vector ${\text{r}}_{j}$ to a point on the complex unit circle. For a uniform random distribution of atom positions, this mapping will produce points uniformly distributed around the complex unit circle as well. In that case, when summing the exponential term over all sheet atoms, taking the norm, and normalizing by the number of atoms N, this will tend to zero for large number of atoms, i.e., S=0. However, given that the reciprocal lattice vectors per definition satisfy ${\text{a}}_{i}\cdot {\text{b}}_{j}=2\pi \delta_{\mathrm{ij}}$, if an atom coincides with a lattice point, i.e., ${\text{r}}_{j}=n_{1}{\text{a}}_{1}+n_{2}{\text{a}}_{2}+n_{3}{\text{a}}_{3}$ for integers $n_{i}$, the exponent simplifies to $i\text{G}\cdot {\text{r}}_{j}=i\left(n_{1}+n_{2}+n_{3}\right)2\pi$. Thus, we observe that when all atoms perfectly coincide with the substrate lattice, they are mapped to the same point on the unit circle, resulting in S=1. Therefore, the quantity S∈[0,1] will effectively indicate the commensurability between the substrate lattice and the sheet. Note that this quantity is invariant to phase shifts since any translation of the sheet will simply translate the whole set of points along the complex unit circle, which does not alter the norm. Applying the commensurability measure in Eq. **1** to the simulation data of Fig. 2, we obtain the data points shown in blue in the *Bottom* row of Fig. 4. By plotting the friction trend in red for reference (min-max scaled to match the commensurability interval), we find visual consistency between commensurability and friction in response to changes in strain. This indicates that the commensurability is a key reason for the nonmonotonic relationship between the strain on the graphene sheet and the friction force between the sheet and the substrate.

## 5. Sheet-Based Metamaterials

We argue that the strong friction–strain relationship in kirigami graphene sheets can be used to produce a variety of frictional behaviors, including nonmonotonic friction–strain relationships. The friction is not controlled by the contact area between the sheet and the substrate, but rather by their commensurability, which can be controlled by straining the graphene sheet. The combination of a strong friction–strain relationship and a nominally relatively weak friction–load relationship allows the use of load-strain coupling to control the strain, and thereby friction, without introducing significant secondary effects from the increasing load required for strain control. This enables a variety of nanomachine designs, in which the differential friction coefficient is controlled both by the nanomachine coupling and by the kirigami patterning.

We expect that similar mechanisms will be present for other combinations of 2D materials and substrates as long as they are either nominally superlubric or can be made superlubric by kirigami cuts and strain. The details of the frictional response will likely be different, exemplified by the relatively large difference between honeycomb-patterned and tetrahedron-patterned graphene kirigami. This variation represents a wide design space that opens up for new classes of metamaterials with complex and tunable friction properties. Moving beyond 2D, one may imagine that similar cuts in porous 3D materials can produce new and exotic elastic responses and failure properties.

## 6. Materials and Methods

The simulated system consists of a single-layer graphene sheet and a crystalline silicon substrate. The graphene sheet has lateral dimensions of ∼130Å×163Å, containing 8,060 atoms before applying kirigami-styled cuts. The substrate has a thickness of ∼11Å and its lateral size ensures a 10 Å margin in the x-direction and a 15 Å margin in the y-direction between a maximally strained sheet and the substrate edges. For a maximum attempted strain of 2.0, the simulated substrate has an x-y-size of ∼150Å×519Å containing 49,068 atoms, yielding a total of 57,128 atoms in the full system. The graphene sheet is described by the 2D primitive lattice vectors

$$\begin{array}{ccc} & {\text{a}}_{1}^{G}=a^{G}\left(\frac{\sqrt{3}}{2},-\frac{1}{2}\right), & {\text{a}}_{2}^{G}=a^{G}\left(\frac{\sqrt{3}}{2},\frac{1}{2}\right), \end{array}$$

for lattice spacing $a^{G}=2.46\;Å$ and the lattice basis $\left\{\left(0,0\right),\frac{a}{2}\left(1/\sqrt{3},1\right)\right\}$. This corresponds to an atom spacing of ∼1.42Å. The silicon substrate has a diamond structure with the primitive lattice vectors

$$\begin{array}{c} {\text{a}}_{1}^{S}=\frac{a^{S}}{2}\left(\begin{array}{c} 0\\ 1\\ 1 \end{array}\right),\;\;{\text{a}}_{2}^{S}=\frac{a^{S}}{2}\left(\begin{array}{c} 1\\ 0\\ 1 \end{array}\right),\;\;{\text{a}}_{3}^{S}=\frac{a^{S}}{2}\left(\begin{array}{c} 1\\ 1\\ 0 \end{array}\right), \end{array}$$

and lattice spacing $a^{S}=5.431\;Å$. The corresponding reciprocal lattice vectors are given

$$\begin{array}{c} {\text{b}}_{1}^{S}=\frac{2\pi }{a^{S}}\left(\begin{array}{c} -1\\ 1\\ 1 \end{array}\right),\;\;{\text{b}}_{2}^{S}=\frac{2\pi }{a^{S}}\left(\begin{array}{c} 1\\ -1\\ 1 \end{array}\right),\;\;{\text{b}}_{3}^{S}=\frac{2\pi }{a^{S}}\left(\begin{array}{c} 1\\ 1\\ -1 \end{array}\right). \end{array}$$

**Fig. 5.**
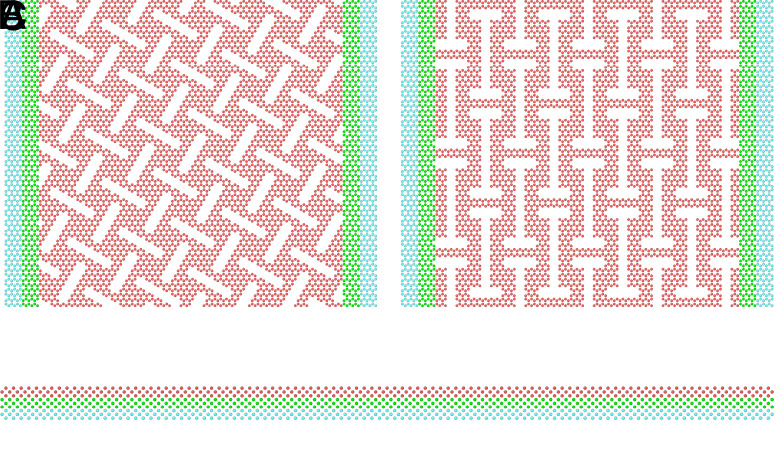
Kirigami patterns: Tetrahedron (*A*) and Honeycomb (*B*), along with a cropped side view of the substrate (*C*). The atoms are colored to indicate the NVE (red), thermostated (green), and rigid (blue) regions of the sheet.

The system is divided into three types of regions as suggested by the color coding in Fig. 5: NVE (red), thermostated (green), and rigid (blue). The NVE region corresponds to a typical MD approach solving Newton’s equation of motion while the thermostated (NVT) regions corresponds to the solving of the Langevin equation to control the temperature. We use a target temperature of T=300K and a damping constant of 1 ps. The rigid regions are treated as rigid objects, meaning that the forces acting on a rigid region are averaged and distributed evenly between all atoms within the region. For the two rigid regions of the sheet, we additionally treat them as a single rigid object with respect to the z-direction, normal to the sheet. This ensures that the rigid ends of the sheet maintain the same elevation relative to the substrate. For the substrate, we let the rigid region be fixed in space as well to avoid any drift of the system as a whole. We refer to the combined thermostated and rigid regions of the sheet as the handling regions which are used for straining, loading, and sliding the sheet. The remaining NVE region of the sheet serves as a canvas for the kirigami patterns, which are created by removing atoms from the sheet. The realization of the two kirigami patterns under study is shown in Fig. 5, with the Tetrahedron pattern (*A*) and the Honeycomb pattern (*B*), along with a cropped view of the substrate (*C*). For the modeling of the interatomic forces, we use a Tersoff potential (31) for the graphene sheet (C–C) with parameters from ref. 43, a Stillinger–Weber potential (32) for the silicon substrate (Si–Si) with parameters from ref. 32, and a Lennard–Jones potential for the interactions between the graphene sheet and the silicon substrate (C–Si), with parameters from ref. 21, with σ=3.0Å and ϵ= 0.0092 eV. The equations of motion are integrated using the Velocity–Verlet scheme with a timestep of 1 fs. The simulation cell has periodic boundary conditions in the lateral directions in order to mimic an infinite substrate. We have chosen the spatial configuration of the sheet such that the sheet does not interact with itself through the periodic boundaries. The system was subjected to the following procedure: The system is initially relaxed for 15 ps. In this period, hard spring-like forces, with a spring constant of 10^5^eVÅ^−2^, are used to fix the sheet centers of mass and restrict rotation of the sheet. The rigid regions of the sheet are temporarily allowed to move freely in the x-y-plane facilitating an equilibration of interatomic distances to account for thermal expansion. The sheet is then strained by a linear translation of the handling regions in the ±y direction with a strain rate of 0.01 ps-1 until a specified uniaxial strain level is reached. The fully strained sheet is relaxed for 5 ps before loading the handling regions uniformly in the direction normal to the sheet and substrate, with a specified normal load $F_{\text{N}}$. During the first 0.5 ps after applying load, we enforce a viscous damping force, F=−γv, with γ=0.05p sÅ, in order to avoid a hard impact between the sheet and substrate. For a remaining 4.5 ps, we let the system relax in the established contacting state. Finally, the sheet is slid in the positive y-direction by translating the handling regions linearly, in a rigid fashion, at a constant speed of 20 ms^−1^ for 2 ns, so that it slides 40 nm in total. For the coupled simulations, we modify the simulation procedure slightly. We apply a sheet tension force on the handling regions with opposite directions, pulling them away from each other, and with a magnitude proportional to the applied sheet load, $F_{t}=T\cdot F_{N}$, with a load-to-tension ratio T=1. Consequently, the handling regions move apart until the in-plane sheet stress balances with the applied tension force. Therefore, we can no longer translate the handling regions in a strictly linear manner, as this would necessitate a fixed distance from each other. Instead, we translate the sheet by applying a spring-like force on the handling regions, based on the distance between the sheet center of mass and a virtual atom moving at the steady translation speed. By choosing a high spring constant, 10^4^ eVÅ^−2^, we achieve a quasi-linear translation which effectively approximates the conditions of the previous simulation procedure.

## Data Availability

Code has been deposited in Zenodo (DOI: 10.5281/zenodo.14105216) (35): Strong strain dependence of friction in graphene kirigami allows engineering a negative coefficient of friction.
